# A Human Taste for Rarity Spells Disaster for Endangered Species

**DOI:** 10.1371/journal.pbio.0040439

**Published:** 2006-11-28

**Authors:** Liza Gross

The shady pursuit of endangered bird eggs made international headlines in May 2006 when Colin Watson, widely considered Britain’s most notorious illegal egg collector, died after falling from a 12-meter tree, allegedly while hunting a rare egg. (Watson’s son Kevin has publicly claimed that his father hadn’t collected an egg since the practice was banned in 1985.) The Royal Society for the Protection of Birds estimates that up to 30 of Britain’s most vulnerable species are targeted by collectors.

Classical economics theory predicts that such exploitation is unlikely to extinguish a species because the cost of finding the last individuals would outweigh the benefits. But a new theoretical study shows that adding human behavior to the equation—specifically, the human penchant for rarity—reveals an unexpected mechanism of exploitation, with alarming implications for species survival. Franck Courchamp, Elena Angulo, and their colleagues incorporated the assumption that rarity increases a species’ value into a classic model of resource exploitation used to manage fisheries. Prizing rarity, they found, triggers a positive feedback loop between exploitation and rarity that drives a species into an extinction vortex.

This phenomenon, the authors explain, resembles an ecological process called the Allee effect, in which individuals of many plant and animal species suffer reduced fitness at low population densities, which increases their extinction risk. Reduced survival or reproduction can occur if individuals fail to find mates, for example, or suffer increased mortality by losing the benefits of pack hunting (more access to prey) or foraging in groups (minimized predation risk). Most studies assume the Allee effect is an intrinsic species trait that human activity cannot artificially induce. But the authors’ model shows that humans can trigger an “anthropogenic Allee effect” in rare species through a paradox of value. When rarity acquires value, prices for scarce species can skyrocket, even though continued exploitation will precipitate extinction.

The model predicts that as long as there is a positive correlation between a species’ rarity and its value, and the market price exceeds the cost of harvesting the species, harvesting will cause further declines, making the species ever rarer and more expensive, which in turn stimulates even more harvesting until there’s nothing left to harvest. And as long as someone will pay any price for the rarest of the rare, market price will cover (and exceed) the cost of harvesting the last giant parrot, tegu lizard, or lady’s slipper orchid on Earth.

The authors describe multiple human activities that could precipitate the anthropogenic Allee effect. Hobby collections of the sort Watson allegedly gave his life for top their list. Overhunting for food and feathers pushed the great auk *(Pinguinus impennis)*—a flightless, now-extinct bird that laid only one egg a year—to the brink of extinction. But it was likely scientists and museum collectors anxious to nab an increasingly rare specimen, the authors suggest, that finished the bird off. And trophy hunting collectors have placed increasing pressure on rare species as their focus has shifted from killing the most dangerous animals to killing the rarest.

**Figure:**
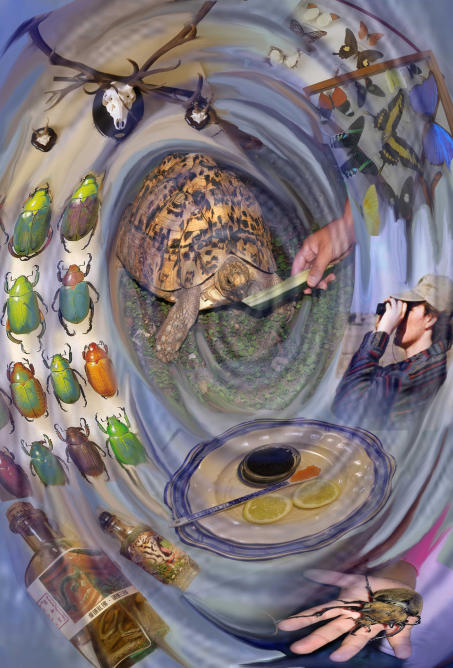
By placing an exaggerated value on rarity, humans can drive rare species into a vortex of extinction, through a process called the anthropogenic Allee effect. (Image: Maria Angulo)

The pursuit of social status and health can also trigger the anthropogenic Allee effect, as many rare species are coveted as luxury items—whether for handbags, exotic cuisine, or dining room furniture—or traditional medicines. The exotic pet trade continues to threaten orangutans, monkeys, reptiles, birds, and wild cats, as well as a wide variety of arachnids, insects, and fish. The great majority of targeted animals die during capture or transport and never even reach the consumer. And it appears that pet trade dealers read the scientific literature for clues to the next hot species: immediately after an article recognized the small Indonesian turtle *(Chelodina mccordi)* and Chinese gecko *(Goniurosaurus luii)* as rarities, their prices soared. The turtle is now nearly extinct and the gecko can no longer be found in its southeastern China niche.

Even well-intentioned activities like ecotourism can destabilize threatened populations. A recent study of killer whales in the North Pacific found an inverse relationship between the number of whale-watching boats one year and a reduced whale population size the next, in keeping with evidence that motorized boats can lower whale fitness. The study also found that the smaller population size one year didn’t discourage whale watching tours the next year, but stimulated interest, based on the larger number of boats.

How to conserve biodiversity when simply declaring a species endangered catalyzes its exploitation? Since many collectors, pet owners, and ecotourists actually care about biodiversity, the authors hope that education may go a long way toward curbing these human activities. Education could even mitigate the damage of trophy hunting and luxury consumption if society stigmatized activities responsible for driving a species to extinction and people could no longer take pride in displaying such “treasures.” But for those who prize rarity above all else, only strengthened regulations and interventions will decrease the probability of a coveted species’ extinction. And until those protections are firmly in place and enforceable, biologists may do well to think twice before reporting a species’ decline.

